# *STK11* as an Emerging Biomarker in Non-Small Cell Lung Cancer

**DOI:** 10.3390/curroncol33050241

**Published:** 2026-04-22

**Authors:** Amit A. Kulkarni, Adam Rock, Matthew Lee, Amanda Reyes, Manish R. Patel, Robert A. Kratzke, Ravi Salgia

**Affiliations:** 1 Department of Medical Oncology and Therapeutics Research, City of Hope Phoenix, Goodyear, AZ 85338, USA; 2 Department of Medical Oncology and Therapeutics Research, City of Hope National Medical Center, Duarte, CA 91010, USA; arock@coh.org (A.R.); matthlee@coh.org (M.L.); amareyes@coh.org (A.R.); rsalgia@coh.org (R.S.); 3 Division of Hematology, Oncology and Transplantation, University of Minnesota, Minneapolis, MN 55455, USA; patel069@umn.edu (M.R.P.); kratz003@umn.edu (R.A.K.)

**Keywords:** *STK11*, LKB1, NSCLC, immune checkpoint inhibitors, PD-L1, *KRAS*, *KEAP1*, immunotherapy resistance, metabolic reprogramming, targeted therapy

## Abstract

Immune checkpoint inhibitors (ICIs) have transformed non-small cell lung cancer (NSCLC) treatment; however, durable responses occur in only a subset of patients, underscoring the need for robust predictive biomarkers. Serine/threonine kinase 11 (*STK11*) is an emerging biomarker that portends poor prognosis and predicts therapeutic resistance. Loss of *STK11* disrupts AMPK signaling, leading to unchecked mTOR activation, metabolic reprogramming, angiogenesis, and epithelial–mesenchymal transition, fostering tumor progression and immune evasion. *STK11* mutations frequently co-occur with *KRAS* and *KEAP1* alterations, exhibit low PD-L1 expression, an immunosuppressive tumor microenvironment that leads to the development of PD-1/PD-L1 resistance. Clinical studies consistently demonstrate inferior outcomes with ICIs in *STK11*-mutant NSCLC, particularly in the presence of *KRAS* and *KEAP1* co-mutations. Dual checkpoint inhibition combining PD-1/PD-L1 and CTLA-4 blockade shows promise in overcoming resistance, results remain inconsistent, and prospective trials are ongoing. Beyond immunotherapy, *STK11* mutations confer poor outcomes across targeted therapies, including *KRAS* G12C inhibitors, with *KEAP1* co-mutation serving as a strong negative predictor of efficacy. In this review we present an overview of *STK11* function and its role in tumor biology, highlight the prognostic and predictive potential of *STK11* mutations in the context of NSCLC treatment and summarize the emerging treatment strategies.

## Simple Summary

Non-small cell lung cancer (NSCLC) is by far the most common cause of cancer related deaths in the United States. Researchers increasingly recognize that certain genetic changes in tumors can influence how well treatments work. One important gene, *STK11*, is often altered in lung adenocarcinoma and is linked to faster tumor growth and less likely to benefit from standard immunotherapies. When *STK11* mutations occur together with other gene alterations, particularly *KRAS* or *KEAP1*, the disease can become even harder to treat. Scientists are exploring new approaches to help these patients, including combinations of immunotherapies and treatments that target cancer metabolism or repair pathways. Understanding the role of *STK11* in lung cancer may help improve treatment selection and guide the development of more effective therapies for patients with these challenging cancers.

## 1. Introduction

Non-small cell lung cancer (NSCLC) is by far the leading cause of cancer-related deaths in U.S. [1]. Immune checkpoint inhibitors (ICI) targeting CTLA-4/PD1/PD-L1 have revolutionized the therapeutic landscape of NSCLC offering unprecedented survival benefits over traditional cytotoxic chemotherapies [2]. ICI are the backbone of treatment in patients with NSCLC without actionable genomic alterations across the spectrum of disease stages [2]. However, a major limitation of ICI therapy is that only a fraction of patients has durable clinical benefit. A large majority of patients either develop primary or secondary resistance to ICI [3]. The Society for Immunotherapy of Cancer (SITC) defines primary resistance as progressive disease (PD) or stable disease (SD) lasting less than 6 months following drug exposure for at least 6–12 weeks [4]. Secondary resistance is defined as best response of complete response (CR), partial response (PR), or SD lasting for 6 months or more following drug exposure of more than 6 months [4]. PD-L1 expression by immunohistochemistry (IHC), is primarily the only biomarker used in clinical practice to predict ICI benefit, but it has important caveats: Definition for high/low expression are arbitrary, degree of responses vary widely among patients with high PD-L1 levels, and some patients with low or undetectable expression still benefit [5]. This inconsistency may be related to tumor heterogeneity, dynamic expression of PD-L1 over time, and assay variability, as well as the influence of tumor microenvironment and host immunity. More reliable biomarkers to optimize patient selection and outcomes of ICI in NSCLC are needed.

Adoption of next generation sequencing (NGS) and novel multi-omics technologies have led to better understanding of the molecular underpinnings of biologic and treatment response heterogeneity. In this context, *STK11* has emerged as an important biomarker for predicting resistance to ICI [6,7,8]. Disruption of *STK11* function can lead to unchecked cell proliferation, metastatic dissemination, lower tumor PD-L1 expression, and an immunosuppressive microenvironment leading to poor treatment response to ICI [9].

In this article, we aim to provide a comprehensive review supporting the role of *STK11* as a biomarker in NSCLC, encompassing the disease biology, tumor suppressive function, co-mutation landscape, and impact on treatment outcomes in the context of immunotherapy and targeted therapy treatment. We review the ongoing clinical trials focused on *STK11* mutant NSCLC and discuss emerging treatment strategies.

## 2. STK11 Mutations and Co-Mutation Patterns in NSCLC

*STK11* is the third most frequently mutated (missense or non-synonymous mutations) gene in lung adenocarcinoma seen in up to 15–33% of cases, following *KRAS* and *TP53* [10]. It is less common in squamous cell histology with reported incidence of 1–5% [11,12]. *STK11* mutations are enriched in Caucasians and African Americans (15–17%) and less common in Asian populations (4–7%) likely due to differences in smoking rates and germline mutational background [13]. *STK11* mutations are typically seen in older patients and are strongly associated with tobacco exposure [14]. The mutational signature is linked to tobacco carcinogen exposure (C>A transversions). No strong sex association is reported. Approximately, 400 unique mutations have been described in *STK11* [15]. Common mutation types include frameshift mutations in hotspots (Q37X, 837–842delC, codons 51–53), truncating mutations, and missense mutations [16]. Mutations in exons 1 and 2 tend to be more disruptive than exons 3–9 [17].

*STK11* mutations frequently occur as co-mutations with *KRAS*, *TP53*, *CDKN2A*, and *KEAP1*. Concurrent co-mutations with *KRAS* are the most common (30–50%), followed by *TP53*, *CDKN2A*, and *KEAP1* [18,19]. *KEAP1* (Kelch-like ECH-associated protein 1) encodes a protein that is crucial for the proteasomal degradation of the nuclear factor erythroid-2 related factor 2 (NFE2L2/NRF2), a transcription factor important in controlling the cellular response to oxidative stress [20,21]. Like *STK11*, *KEAP1* also resides in the chromosome 19p region and is frequently co-mutated with *KRAS* (40–47%) and *STK11* (28%) [22]. In one study, ~ 90% of both *STK11* and *KEAP1* co-mutant tumors had evidence of the loss of heterozygosity (LOH) by biallelic inactivation [23]. Triple mutant *STKL11*/*KEAP1*/*KRAS* is rare but is associated with worse prognosis. Finally, the loss of *STK11* expression by IHC in the absence of *STK11* mutations (or *STK11* wild type) is common (17.6%) in the context of *KRAS* mutations [8].

## 3. STK11 Biology

*STK11*, also known as liver kinase B1 (LKB1) belongs to the calcium calmodulin family, that is ubiquitously expressed and is highly conserved [24]. It was initially studied in the context of Peutz–Jeghers syndrome, an autosomal dominant disorder characterized by mucocutaneous melanin pigmentation, gastrointestinal hamartomatous polyposis, and an increased risk of developing various neoplasms [25]. *STK11* is a serine/threonine kinase located on chromosome 19p13.3, spanning approximately 23 kb of genomic DNA, and consists of 9 exons encoding a 433 amino acid [26]. The N-terminal domain of the serine/threonine kinase contains a nuclear localization signal that is essential for catalytic activity and binding downstream substrates; a central kinase domain (residues 44–39) and the C-terminal regulatory region stabilizes and activates the kinase [27,28]. The active form of *STK11* is part of a complex with the pseudokinase STe20-Related ADaptor protein (STRAD) and the armadillo repeat-containing mouse protein 25 (Mo25) [9]. Formation of this complex regulates the stability, the subcellular localization, and the kinase activity of *STK11*. *STK11* acts as a master regulator of diverse cellular functions including energy homeostasis, cell polarity, chromatin remodeling, and is an important tumor suppressor gene. *STK11* regulates the activity of at least 14 downstream kinases related to the *AMPK* family and phosphorylates other substrates including STRAD, PTEN, and p21CDKN1A [29,30].

### 3.1. Preclinical Models of STK11 as a Regulator of Cellular Metabolism, Growth and Polarity

**Figure 1:**
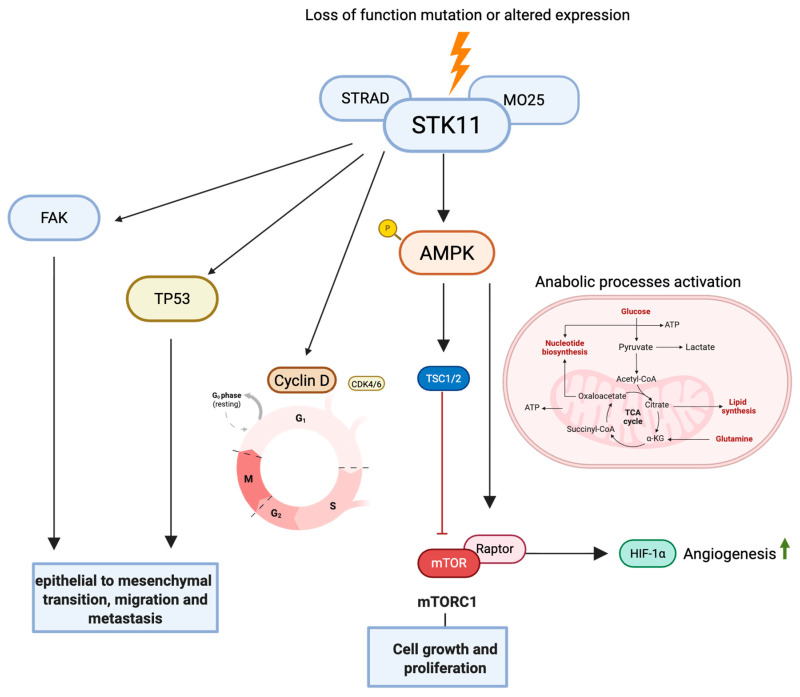
Summarizes the effects of the loss of STK11 function as a tumor suppressor gene. STK11 loss leads to disrupted AMPK activation, leading to unchecked mTORC1 signaling and increased anabolic metabolism. HIF-1α–mediated angiogenesis alter Cyclin D regulation, and interacts with FAK and TP53 pathways to drive epithelial–mesenchymal transition (EMT), migration, and tumor proliferation. Created in Biorender. Kulkarni, A. (2026) (https://app.biorender.com/illustrations/693208f644b8034b16b1d069) (accessed on 6 February 2026).

The role of *STK11* in carcinogenesis is complex and functions as a tumor suppressor gene and oncogenic gene through multiple putative mechanisms (Figure 1). *STK11* serves as a central metabolic “energy sensor” to conserve ATP through direct phosphorylation and the activation of AMP-activated protein kinase (AMPK) [31,32,33]. Loss of *STK11* disrupts the activation of *AMPK*, leading to the unchecked activation of the often called (and originally identified as) mammalian target of rapamycin (mTOR) driving anabolic metabolism and cell proliferation. *STK11*-deficient tumors exhibit increased reliance on glycolysis and glutaminolysis reflecting adaptive metabolic reprogramming under metabolic stress [34,35]. This metabolic reprogramming not only supports rapid tumor growth and survival but also confers resistance to agents targeting the PI3K/AKT/mTOR axis, as feedback loops and compensatory pathways become dysregulated in the absence of *STK11* function. 

*STK11* suppresses hypoxia-inducible factor alpha (HIF1A) thereby reducing angiogenesis; its loss promotes hypoxia-driven tumor progression [36]. *STK11* controls cell cycle through the transcriptional regulation of Cyclin D1 and p21CDKN1A [37,38]. *STK11* is essential for the establishment of epithelial cell polarity through the regulation of PAK1 and the modulation of the phosphorylation status of FAK and CDC42 activation [39]; its deficiency disrupts tissue architecture and facilitates cellular proliferation. Mechanistic studies show that *STK11* loss drives epithelial–mesenchymal transition (EMT) and metastatic potential [15,40].

Lastly, in models of genotoxic stress, *STK11* interacts with p53 and DNA damage response (DDR) proteins to promote apoptosis [41,42] contributing to genomic instability and resistance to DDR-dependent strategies [43]. Taken together, these changes can impair drug delivery and penetration, reduce the effectiveness of anti-angiogenic agents, and promote survival signaling that counteracts the intended effects of targeted therapies.

### 3.2. Preclinical Data Underlying STK11 Loss Induced Immunosuppression

Loss of *STK11* induces a pro-inflammatory but immunosuppressive tumor-immune milieu with increased pro-inflammatory cytokine production, altered angiogenesis, and recruitment of immunosuppressive myeloid cells, all of which contribute to a more hypoxic, nutrient-deprived, and drug-resistant microenvironment [9,44].

*STK11* loss represses STING expression via DNMT1 and EZH2-mediated epigenetic silencing, reducing type I interferon signaling and impairing T-cell recruitment [45]. Importantly, studies show that *STK11* functional loss—without mutation—is sufficient to trigger immune dysfunction through this axis [46].

*STK11*-loss lung adenocarcinomas display global hypomethylation, SAM-e depletion, downregulation of DNMT1, and increased expression of repetitive genomic elements—contributing to transcriptional instability and an altered immune landscape [47].

In mouse *KRAS*-mutant NSCLC, *STK11* loss increases neutrophil infiltration via: CXCL7, CXCL3, CXCL5 chemokines, and IL-33 and IL-1α [48], leading to the suppression of CD8+ T-cell infiltration in vivo [44]. *KEAP1*-deficient tumors have fewer CD8+ cytotoxic T cells but a retention of or increase in CD4+ T cell subsets, including T helper 1 (TH1) cells (T-bet+ CD4+), resulting in a significantly increased TH1/CD8+ ratio [49].

### 3.3. Clinical Observations Related to Histologic Features, PD-L1 Expression, Tumor Mutational Burden, and Tumor Immune Microenvironment (TIME)

Patients with STK11 mutant lung adenocarcinomas have solid and granular adenocarcinomas morphology with robust HepPar1 expression but without any expression of any other hepatocellular markers on immunohistochemistry [50]. These tumors had mitochondria-rich cytoplasm which may represent a metabolic adaption in response to STK11 loss and exhibit an exceptionally aggressive clinical behavior.

Patients with *STK11*/*KEAP1*-mutant NSCLC demonstrate higher tumor mutational burden (TMB) (median TMB 5.22 versus 13.05 mut/MB) [48] but paradoxically lower PD-L1 expression compared with wild-type tumors [51,52,53]. Across clinical studies, *STK11*-mutant tumors consistently show reduced or absent PD-L1 expression, independent of *KRAS* mutation status [22,51,52,53,54,55]. In *KEAP1*-mutant but *KRAS*-wild-type tumors, PD-L1 suppression appears less pronounced, suggesting interaction between *KRAS* and *KEAP1*/*STK11* biology.

While *STK11* and *KEAP1* are frequently co-mutated, they have a slight differential impact on the immune cell subsets in the tumor immune microenvironment (TIME). *STK11* inactivation predominantly boosts the neutrophil/CD8^+^ ratio, whereas loss of *KEAP1* leads to a TIME rich in tumor-associated macrophages and monocytes as well as neutrophils [48]. Specifically, m*STK11* tumors have a profound depletion of CD8+ cells and relative retention of CD4+ T cells, resulting in an increased TH1/CD8+ ratio [48].

## 4. STK11 as Prognostic and Predictive Biomarker

### 4.1. Prognostic Relevance of STK11 in Patients with NSCLC Treated with Surgery, Chemotherapy and/or Radiotherapy

Real-world data suggests that mutant *STK11* (m*STK11*) NSCLC have higher rates of recurrence and inferior survival outcomes across all stages, patient populations, and treatment modalities (Table 1). A meta-analysis of patients with NSCLC across all stages and treatments (not including immunotherapy) showed that m*STK11* was associated with worse progression free survival (PFS) (Hazard ratio (HR) = 1.69, 95% confidence interval (CI) 1.16–2.45) and overall survival (OS) (HR = 1.50, 95% CI 1.01–2.24) compared to wild-type *STK11* (wt*STK11*) [56]. In a Chinese study of patients with stage I-III NSCLC (N = 447) treated with surgical resection and adjuvant treatment, m*STK11* was associated with worse OS (*p* = 0.031) in patients with stage III NSCLC but was not significant on multivariable regression analysis [57]. In a Cancer Genome Atlas (TCGA) cohort of NSCLC adenocarcinoma (N = 421), patients with m*STK11* (n = 67) had inferior OS [HR 3.36 (95% CI: 1.23 to 9.21), *p* < 0.05] when compared to wt*STK11*. Co-mutation with *KRAS* was equally detrimental to OS [HR 3.37 (95% CI: 1.33 to 8.49) [10].

A pooled cohort of patients with unresectable early-stage NSCLC (Stage I-II) receiving definitive radiation (N = 62), when compared to wt*STK11* patients with m*STK11*, was associated with statistically significant lower 2-year disease free survival (DFS) rates (71% vs. 30.8%; HR 6.8 (95% CI 2.50–18.3; *p* = 0.0002). Despite the effective local control, *mSTK11* had lower 2-year OS rate (52% versus 85%; HR 6.0 (95% CI 1.30–27.80; *p* = 0.022). Furthermore, m*STK11* tumors were associated with higher incidence of distant failure than local recurrence (40.4% vs. 12.1%) [58]. In another study of patients with stage III NSCLC (N = 164) a large majority of whom received chemoradiation, m*STK11* patients were associated with higher rates of locoregional recurrence (25% vs. 10.8%), shorter DFS [HR 2.53 95% CI 1.37–4.65; *p* = 0.002] and inferior OS [HR 2.19 95% CI 1.6–4.25; *p* = 0.033] when compared to wt*STK11* [59]. Similarly, a study of patients with stage III NSCLC treated with concurrent chemoradiation, PFS was inferior in the m*STK11* versus wt*STK11* (HR = 2.25; 95% CI, 1.03–4.88, *p* = 0.04), whereas OS was numerically lower but not statistically significant (HR 1.47, 95% CI, 0.49–4.38, *p* = 0.49) [60].

The type of mutation and mutational context also appears to determine prognosis. For instance, stratification by co-mutations with *KRAS*/*KEAP1* and the location of mutation appear to have a differential outcome. In one study of patients with resected NSCLC (N = 567), patients with m*STK11* in exons 1–2 had a lower OS when compared to m*STK11* in exons 3–9 (median OS 24 months versus 91 months; log-rank, *p* = 0.003) or wild-type (24 months vs. 69 months; log-rank, *p* = 0.005). The difference (m*STKL11* exons 1–2 vs. wt*STK11*) was statistically significant (*p*= 0.002). On the other hand, there was no difference in OS for patient with *STK11* exons 3–9 mutation as compared to wt*STK11* patients (log-rank, *p* = 0.29) [17].

Similarly, co-mutation with *KRAS* appears to have a worse prognosis. A large observational study in patients with metastatic NSCLC treated with first-line chemotherapy (N = 2137) showed worse PFS and OS outcomes in patients with m*STK11* versus wt*STK11* (for both PFS and OS- HR, 1.4 [1.2–1.6; *p* < 0.0001]. Co-mutation with *KRAS* were associated with even worse OS and PFS outcomes compared to wt*KRAS* [HR for OS 1.6 (1.3–1.9); HR for PFS 1.4 (1.2–1.7)] [61].

Lastly, a computational tool for survival risk stratification and biomarker identification using sequencing data in a cohort of advanced lung adenocarcinomas (N= 1054) found that *STK11* and *KEAP1* co-mutations were the strongest determinants of poor prognosis (median OS of 7.3 months versus 32.8 months; HR 4.6 (*p* < 0.001) when compared to demographic and other genomic predictors (*TP53*, *KRAS*, etc.) [23].

### 4.2. Prognostic Impact of STK11 on Immunotherapy or Chemoimmunotherapy Outcomes

ICI alone or in combination with chemotherapy are United States (U.S.) Federal Drug Administration (FDA) approved for the first-line treatment of locally advanced or metastatic NSCLC in the absence of actional genomic alterations across the whole PD-L1 spectrum [62]. There is a positive correlation between the level of PD-L1 expression and ICI benefit [63]. In patients with high PD-L1 expression (PD-L1 tumor proportion score (TPS) ≥ 50%), immunotherapy alone (PD-L1/PD-1) is reasonable, however, combining with chemotherapy can be considered in select patients based on patient characteristics (level of PD-L1 expression, performance status, disease burden, smoking history), and personal preferences. In patients with low PD-L1 (PD-L1 expression TPS 1–49%) and PD-L1 negative (PD-L1 < 1%), chemoimmunotherapy is favored over immunotherapy alone [62]. The presence of *STK11* mutation status may guide the decision for optimizing treatment selection in metastatic NSCLC regardless of the level of PD-L1 expression. Data from randomized clinical trials (RCT) and real-world studies suggest the negative prognostic impact of *STK11* mutation in patients treated with ICI and co-mutation with *KRAS* and *KEAP1* may further erode this clinical benefit (Table 2). In exploratory analysis of KEYNOTE-189 study, clinical benefit of pembrolizumab with chemotherapy (objective response rate (ORR), PFS and OS) was lower in patients harboring m*STK11* and m*KEAP1,* but formal statistical comparison was unavailable [53]. On the other hand, in the KEYNOTE-042 study, clinical outcomes (ORR, PFS, and OS) with pembrolizumab were similar in NSCLC patients with or without mutant *STK11* or *KEAP1* [52]. Similarly, in another study (N = 574) of patients NSCLC treated with first-line ICI *STK11* and *KEAP1* mutations were associated with poor prognosis, but were not predictive of ICI benefit [64]. The inconsistency across the studies needs to be interpreted cautiously since concurrent *KRAS* mutation status was unknown, and the treatment groupings were heterogeneous amongst the studies.

The prognostic and predictive potential of *STK11* appears to be more consistent in *KRAS* mutant cancers than wild type *KRAS*. Several prospective and real-world studies have shown the detrimental impact of *STK11* mutation on ICI outcomes in the context of *KRAS* co-mutation in NSCLC patients. In a CheckMate-057 study, in patients with co-mutated *KRAS* and *STK11* NSCLC, the ORR was 0% in both the nivolumab and docetaxel arms [8]. Post hoc analysis (N= 1202) from a IMpower150 study (atezolizumab plus bevacizumab plus carboplatin/paclitaxel (ABCP) or atezolizumab plus carboplatin/paclitaxel (ACP) or bevacizumab plus carboplatin/paclitaxel (BCP)) showed that *STK11* and *KEAP1* mutations were associated with overall inferior PFS and OS; and patients with *STK11*/*KEAP1* co-mutation had the worst prognosis [55]. As opposed to wild type *KRAS*, patients with mutant *KRAS* and co-mutated *STK11* and/or *KEAP1* tumors appear to be predictive of OS and PFS benefit from the ABCP regimen. For instance, in *KRAS* mutated patients, co-mutation with *STK11* and/or *KEAP1*, led to longer OS in the ABCP arm (median 11.1 months; HR 0.60; 95% CI 0.34 to 1.03) than in the ACP arm (median, 7.9 months; HR 0.87; 95% CI 0.52 to 1.45) versus the BCP arm (median 8.7 months). However, in patients with wild type *KRAS*, co-mutation with *STK11* and/or *KEAP1* was not predictive of OS benefit with the ABCP regimen (median 13.2 months; HR 1.04; 95% CI 0.66 to 1.64) or ACP (median, 9.0 months; HR 1.39; 95% CI 0.83 to 2.33) versus BCP (median 12.5 months). A similar trend was also noted with PFS benefit. In another retrospective study, in patients with NSCLC (N = 1261) treated with immunotherapy, *STK11* and *KEAP1* mutations were associated with lower ORR (*STK11* 11.6% vs. 32.4%; *KEAP1* 17.8% vs. 29.3%), lower PFS [*STK11* HR 2.04, *p* < 0.0001; *KEAP1* HR = 2.05, *p* < 0.0001), and lower OS (*STK11* HR = 2.09, *p* < 0.0001; *KEAP1* HR = 2.24, *p* < 0.0001) among *KRAS* mutant patients but not with wild type *KRAS*. Both *STK11* and *KEAP1* mutation were independent predictors of shorter PFS and OS to ICI on multivariable analysis [7].

Skoulidis et al. evaluated *KRAS* mutant tumors for the efficacy of ICI (N= 174) in patients with or without *STK11* mutations [8]. ORR was only 7.4% in *STK11* co-mutated tumors as opposed to *KRAS* only mutant tumors (28.6%). Furthermore, PFS (*p* < 0.001) and OS (*p* = 0.0015) were also significantly shorter in the same population. The impact of *STK11* mutations on clinical outcomes with ICI was more pronounced in PD-L1 negative NSCLC. Another recent study by Skoulidis et al. (N = 871) showed that in patients treated with pembrolizumab and chemotherapy, patients harboring *STK11* and *KEAP1* mutations were independently associated with inferior PFS and OS outcomes irrespective of *KRAS* mutation, TMB and PD-L1 expression [32]. However, the adverse prognosis of m*STK11* mutations appeared to be more impactful only in the presence of concurrent *KEAP1* mutations. In the same token, several other studies also showed the negative impact of SKT11 mutations on ICI clinical outcomes in the context of *KRAS* mutations [36,37,42,64,65,66].

In summary, it is important to contextualize the prognostic and predictive impact of *STK11* mutation on ICI outcomes based on the presence of *KRAS* co-mutations since they are frequently co-mutated and there appears to be a differential impact of *STK11* mutations based on *KRAS* co-mutation status. Furthermore, all these analyses were exploratory, and therefore should be interpreted with caution.

### 4.3. Benefit of Doublet Immunotherapy (CTLA-4+PD-1(L1) Blockade

*STK11*-mutant tumors are associated with an adverse TIME characterized by a preponderance of suppressive myeloid cells, CD8+ cytotoxic T cell depletion, with relative sparing of CD4+ effector cells [48]. This results in reduced PD-L1 expression and diminished T-cell infiltration limiting the efficacy of conventional immunotherapeutic agents targeting the PD-1/PD-L1 axis. Cytotoxic T-lymphocyte-associated antigen 4 (CTLA-4) is an immune checkpoint receptor on T-cells acting as a brake for T-cell activation. CTLA-4 has a higher binding affinity for CD80/CD86 ligands on antigen presenting cells. By outcompeting with CD28 for CD80/CD86 binding, it denies CD28 the co-stimulation necessary for T-cell activation [67]. Therefore, CTLA-4 inhibition may augment the efficacy of immunotherapeutic approaches in *STK11*-mutant disease. In preclinical and clinical studies, dual ICI blockade resulted in a robust increase in CD4+ subsets, including TH17 cell subsets, TH1 T cells (T-bet+CD4+), and effector memory CD4+ T cells (CD4+CD44+CD62L) [68]. In mouse models, dual blockade activated the CD4+ Foxp3, and modulated the myeloid compartment, including the activation of conventional CD103+ dendritic cells (DC) and the expansion of a myeloid subset that produces TNFα and iNOS (TIP-DCs) [69].

Available clinical data suggests an incremental benefit of adding CTLA-4 inhibitors to PD-1/PD-L1 inhibitors in patients with m*STK11*, and low or negative PD-L1 expression, but not in high-PD-L1 expression [70,71,72]. This benefit seems to be more apparent when using the combination of CTLA-4 inhibitors and PD-1/PD-L1 inhibitors with chemotherapy but not in regimens containing immunotherapy doublets without chemotherapy.

Post hoc analysis of the POSIDEON trial has demonstrated the relative benefit of dual PD-L1/CTLA4 inhibition as opposed to PD-L1 therapy alone. In *STK11*/*KEAP1* patients, ORR were higher in those receiving both PD-L1 and CTLA4 inhibition along with chemotherapy as opposed to chemotherapy or chemotherapy/PD-L1 inhibition (42.9% vs. 30.2% vs. 28%, respectively) [48]. Most importantly, dual checkpoint inhibition led to improved PFS and OS irrespective of TMB, *KRAS* co-mutation, or PD-L1 expression [73,74]. Similarly, the CheckMate 9LA study, which compared nivolumab plus ipilimumab plus two cycles of chemotherapy versus chemotherapy alone, recapitulated a PFS benefit with a dual checkpoint blockade in *STK11* and *KEAP1* mutant NSCLC. Given the trial designs, it remains unclear what proportion of this benefit is driven specifically by CTLA4 inhibition [75].

However, other analyses have failed to demonstrate this benefit questioning the contribution of CTLA4 inhibition. The MYSTIC trial, which investigated the combination of durvalumab with or without tremelimumab versus chemotherapy in first-line, metastatic NSCLC, dual checkpoint inhibition failed to demonstrate a significant difference in OS in patients with *STK11*-mutant NSCLC [76]. In the CheckMate 227 trial (nivolumab+ipilimumab versus nivolumab alone versus chemotherapy), there was significant PFS and OS benefit favoring the CTLA-4/PD-1 combination in m*KEAP1* but no such trend was noted in m*STK11* patients. (PFS: 11.1 versus 2.9 months, HR: 0.25 [95% CI: not reported]; OS: 24.4 versus 8.9 months, HR: 0.31 [95% CI: 0.14–0.70]) [70,77].

In summary, the role of CTLA4 inhibition is a promising but ultimately unclear approach to *STK11* and *KEAP1* mutant NSCLC. The ongoing clinical trial, TRITON, seeks to conclusively answer this question in a phase III, randomized fashion (NCT06008093).

## 5. Resistance to Targeted Therapy in STK11-Mutant NSCLC

### 5.1. Non-KRAS-Targetable Mutations

Patients with m*STK11* NSCLC have worse PFS and OS following treatment with all therapies including targeted therapies compared to *STK11* wild-type patients. In a meta-analysis of 4317 NSCLC patients, including 605 with m*STK11*, the PFS and OS HRs were 1.49 and 1.44 respectively, indicating a substantial negative impact on outcomes [56]. This effect is observed across all forms of systemic therapy, including *EGFR*, *ALK*, *KRAS*, *MET*, *RET*, *ROS1*, *BRAF*, and *HER2* inhibitors [78,79].

For EGFR-mutant NSCLC, *STK11* mutations are usually mutually exclusive with EGFR mutations, but when present, they are associated with poor response to EGFR tyrosine kinase inhibitors (TKIs) such as osimertinib. In a cohort of 960 patients with metastatic EGFR-mutant lung adenocarcinoma, those with *STK11* alterations had significantly worse PFS and OS compared to *STK11* wild-type counterparts [80]. Functional studies confirmed that *STK11* loss promotes resistance to osimertinib, and that MEK inhibition (trametinib) may partially restore sensitivity in *STK11*-deficient cells [80]. Lastly, cumulative evidence indicates that the presence of rare co-mutations such as *SMARCA4*, *NFE2L2*, and *PTEN* in m*STK11*-mutant NSCLC is associated with a further reduction in objective response rates, PFS, and OS following treatment with targeted therapies [6,81,82,83,84,85]. For *ALK*, *MET*, *RET*, *ROS1*, *BRAF*, and *HER2* inhibitors, direct clinical trial data on the impact of *STK11* mutations are limited, but meta-analyses and real-world studies indicate that the presence of *STK11* mutations confers resistance and poor outcomes across these agents.

### 5.2. KRAS G12C Inhibitors: Predictors and Subgroup Outcomes

*KRAS* G12C inhibitors, such as sotorasib and adagrasib, have become established therapies for *KRAS* G12C-mutant NSCLC. However, even though the presence of an *STK11* mutation in *KRAS* G12C-mutant NSCLC does not preclude response to *KRAS* G12C inhibitors, a co-occurring *KEAP1* mutations and high NRF2 activity are strong negative predictors of efficacy. In the phase 2 CodeBreaK 100 trial of sotorasib, patients with *STK11*-mutant/*KEAP1*-wildtype tumors had an ORR of 50% (95% CI, 28 to 72), compared to 39% (95% CI, 30 to 49) in the overall evaluable population [86]. In contrast, those with both *STK11* and *KEAP1* mutations had lower ORR and shorter PFS and OS [87,88,89,90]. In the single-arm phase 2 KRYSTAL-1 trial of adagrasib (n = 35), the confirmed ORR in patients with co-occurring *STK11* mutations was 30.3%, with median PFS of 4.8 months and median OS of 12.3 months. Median PFS was shorter in patients with concurrent *KEAP1* mutations (5.5 months; 95% CI, 0.5-not estimable [NE]) compared with those without *KEAP1* mutations (n = 21; 8.4 months; 95% CI, 1.4-NE). Whereas patients with mutant *KEAP1*; ORR was 38% vs. 24 in wildtype [91]. High NRF2 signaling, even in the absence of *KEAP1* mutation, was associated with inferior outcomes (PFS 4.2 vs. 8.4 months; OS 6.5 vs. 19.0 months) [87,89]. In summary, *STK11* and *KEAP1*-wildtype patients have response rates comparable to the overall population, while those with both mutations have the poorest outcomes.

## 6. Emerging Therapies in STK11 and KEAP1 Mutations

Targeting *STK11* in NSCLC has presented challenges thus far with several promising compounds failing to show activity (Table 3). Understanding the therapeutic potential of targeting *STK11* and/or *KEAP1* alterations is difficult given the frequent co-expression of oncogenic, driver alterations including *KRAS*.

Bemcentinib, a molecule selectively targeting AXL, previously received breakthrough designation in *STK11*-mutant NSCLC by the FDA. The phase 1b/2a studies of bemcentinib in combination with carboplatin/pemetrexed/pembrolizumab in advanced, metastatic NSCLC stratified by *STK11* alterations (NCT05469178) was terminated due to lack of efficacy. Other combinatorial approaches seek to enhance the responsiveness of *STK11*-mutant NSCLC to conventional immunotherapeutic approaches including targeting ornithine decarboxylase by α-difluoromethylornithine (DFMO) which enhances antitumor CD8+ T cell infiltration [92]. However, DFMO combination with immunotherapy in *STK11*-mutant NSCLC was suspended (NCT06219174). GT103, a fully human, IgG3 monoclonal antibody targeting complement factor that is being deployed in combination with pembrolizumab in *STK11*-mutant NSCLC (NCT07017829), is open for enrollment. Furthermore, the Corepressor of Repressor Element 1 Silencing Transcription (CoREST) complex TNG260, a small molecule inhibitor of the CoRest complex, is being utilized to potentially sensitize SKT11-mutant NSCLC to anti-PD1 based therapies (NCT05887492).

Epigenetic therapies like EZH2 or DNMT1 inhibition inducing STING re-expression may offer ways to overcome resistance. In one study, using MPS1 inhibition primed the immunogenicity of *KRAS*-LKB1 mutant lung cancer [93]. Alternative approaches exploit molecules over-expressed in *STK11*-mutant NSCLC, including CD38. Daratumumab, a human monoclonal antibody targeted CD38, has previously been approved for the treatment of multiple myeloma. Interestingly, *STK11* alterations have been observed to result in an increased CD38 expression [94]. As such, daratumumab is being investigated as a therapeutic approach in *STK11*-mutant NSCLC (NCT05807048).

NRF2 pathway activation routinely seen in *STK11* co-mutation with *KEAP1* appears to rely on glutamine availability. Therefore, glutaminase inhibition is being explored as a potential avenue to overcome the NRF2 upregulation observed in *STK11*-mutant NSCLC. Combination treatment with the glutaminase inhibitor, telagelenstat (CB-839), inhibited clonal expansion and activation of CD8 T cells [95]. Trials like BeGIN (NCT03872427) and KEAPSAKE (NCT04265534) evaluate telaglenastat, aiming to disrupt glutamine metabolism. However, KEAPSAKE study combining telagelenstat with chemoimmunotherapy was terminated due to lack of efficacy. The nuclear factor erythroid 2-related factor 2 (NFE2L2) gene, which is upstream of NRF2, has been observed to upregulate mTOR pathway [96]. As such, TAK-228, a TORC1/2 inhibitor, has been evaluated and noted to have single agent activity in NRF2-activated NSCLC [97]. Unfortunately, a subsequent phase II trial of vistusertib, a selective inhibitor of both mTORC1 and mTORC2, demonstrated low ORR, questioning the future of this approach [98]. Other studies like BUNCH (NCT04518137) using onatasertib, targeting mTOR have also been terminated. Inhibition of the NRF2, *KEAP1*, and Cullin-3 (CUL3) with MGY825 (NCT05275868) was terminated and trial of VVD-130037 (NCT05954312), a *KEAP1* activator, is ongoing.

Given the challenges of inhibiting a mutant protein with loss of function, emerging approaches include proteolysis-targeting chimera (PROTAC) that results in *KEAP1* protein degradation in vitro and vivo [99,100].

## 7. Future Directions

The presence of *STK11* mutations in NSCLC confers resistance to current therapeutics by a variety of mechanisms as described previously. The complex interaction between co-mutations is not fully understood, but the presence of *KEAP1* and *KRAS* co-mutations do appear to have a large impact on the clinical significance of *STK11* mutations including prognostic value and treatment response. It remains a priority to stratify patients with these mutation profiles for treatment escalation, but the optimal treatment escalation is still under investigation including the role of CTLA4 inhibition.

In the current treatment paradigm for *STK11*-mutant NSCLC there are a lack of effective targeted treatments, therefore there is significant interest in novel and investigational therapies. Novel therapeutic options include metabolic targeting, synthetic lethality, and novel combination strategies. Preclinical studies have demonstrated that agents inducing metabolic stress, such as biguanides (metformin, phenformin) can selectively inhibit the growth of LKB1-deficient NSCLC cells [21,101]. Mitochondrial uncouplers, such as piceatannol and tyrphostin 23, have been shown to induce synthetic lethality in *STK11*-deficient tumors by exploiting the HIF1A-LEP-UCP2 axis thereby lowering cellular energy below the threshold for survival, resulting in cell death [102]. Synthetic lethal approaches targeting the YAP/TAZ/TEAD pathway, tRNA-modifying enzymes, and other pathways identified through genome-wide CRISPR screens have shown promise in preclinical models [103,104].

While single agent mTORC1/2 inhibition with vistusertib failed to demonstrate meaningful clinical benefit, preclinical data suggest that rational combinations targeting multiple pathways, such as mTOR, MEK, ERK, and FAK inhibitors, may overcome resistance [21,105]. Nutrient deprivation strategies, including specific dietetic regimens, have also been investigated as alternative therapeutic interventions, with promising results in preclinical studies [21,101]. Preclinical and translational studies support the investigation of metabolic targeting and combination strategies, but clinical data are not yet available [21,102,104,105].

Loss of *STK11* impairs the ability of tumor cells to respond appropriately to genotoxic stress, which can paradoxically increase sensitivity to certain DNA damage response (DDR) targeting agents, such as ATR inhibitors in preclinical models. However, this also confers resistance to therapies that rely on intact apoptotic signaling or cell cycle checkpoints, like ICI [31]. Additional research into these novel therapeutic agents and pathways is needed to determine the effectiveness in the treatment of m*STK11* NSCLC.

## 8. Conclusions

*STK11* alterations in NSCLC have reshaped our understanding of therapeutic resistance and disease biology. These mutations disrupt critical metabolic and signaling pathways, creating a tumor phenotype that is both aggressive and refractory to standard treatments. Their frequent association with *KRAS* and *KEAP1* co-mutations compounds this challenge, driving complex interactions within the tumor microenvironment that limit immune surveillance and blunt the efficacy of ICI. Current evidence underscores the prognostic significance of *STK11*, with consistent correlations to inferior survival across diverse treatment modalities, including chemotherapy, immunotherapy, and targeted agents.

While conventional strategies have yielded modest benefit, emerging approaches targeting metabolic dependencies, epigenetic regulators, and synthetic lethal vulnerabilities offer a promising horizon. Rational combinations that integrate immunotherapy with agents modulating nutrient utilization or restoring innate immune signaling may help overcome resistance. Furthermore, leveraging multi-omic profiling to contextualize *STK11* within broader genomic landscapes will be essential for precision medicine. Ultimately, translating these insights into clinically actionable interventions requires well-designed prospective trials and biomarker-driven frameworks. Addressing the therapeutic gap in *STK11*-mutant NSCLC remains a critical priority to improve outcomes in this biologically distinct and clinically challenging subset.

## Figures and Tables

**Table 1:** STK11 as a prognostic marker in NSCLC patients receiving non- ICI therapies.

Study	N	Treatment Context	*KEAP1* Co-Mutation	PFS/DFS HR (95% CI)	OS HR (95% CI)	STK11 Effect
Xu Ke et al., 2024 (Meta-analysis) [56]	605	All stages/all non-ICI therapies	Not reported	1.69 (1.16–2.45)	1.50 (1.01–2.24)	Negative
Liao H et al., 2021 [57]	447	Stage I–III/surgery + adjuvant	Not reported	NR	1.04 (0.69–1.25), *p* = 0.031 (stage III only; NS on MVA)	Neutral/Mixed
Malhotra J et al., 2022 (TCGA) [10]	67 mSTK11/421 total	All stages/mixed	Not reported (KRAS co-mut 54%)	NR	mSTK11: HR 3.36 (1.23–9.21); KRAS co-mut: HR 3.37 (1.33–8.49)	Negative
Katipally et al., 2023 [58]	62	Unresectable Stage I–II/definitive RT	Not reported	6.8 (2.50–18.3) [DFS]	6.0 (1.30–27.80)	Negative
Sitthideatphaiboon et al., 2021 [59]	164	Stage III/mixed CRT	Not reported	2.53 (1.37–4.65) [DFS]	2.19 (1.6–4.25)	Negative
An J. et al., 2021 [60]	75	Stage III/concurrent CRT	Not reported	2.25 (1.03–4.88)	1.47 (0.49–4.38), *p* = 0.49 (NS)	Neutral/Mixed
Shire et al., 2020 [61]	2137	Metastatic/1L chemotherapy	Not reported	1.4 (1.2–1.6)	mSTK11: 1.4 (1.2–1.6); KRAS co-mut: OS 1.6 (1.3–1.9)	Negative
Shen et al., 2019 [23]	1054	Advanced LUAD/mixed	*STK11* + *KEAP1* co-mut: worst prognosis (median OS 7.3 vs. 32.8 mo)	NR	STK11+KEAP1: HR 4.6 (*p* < 0.001)	Negative

Abbreviations: NSCLC = Non-small cell lung cancer; ICI = Immune Checkpoint Inhibitor; PFS = Progression-Free Survival; DFS = Disease-Free Survival; OS = Overall Survival; HR = Hazard Ratio; CI = Confidence Interval; NR = Not reported; RT = Radiation Therapy; CRT = Chemoradiotherapy; 1L = First Line; LUAD = Lung Adenocarcinoma. Color Legend: 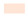 Significant negative effect of STK11 mutation on survival outcome; 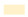 Mixed or partially significant results (e.g., significant in subgroup only).

**Table 2:** Impact of STK11 on Immunotherapy/Chemoimmunotherapy Outcomes in advanced NSCLC.

Study	N	Treatment	*KRAS* Context	*KEAP1* Co-Mutant.	ORR (mSTK11 vs. wt)	PFS HR (95% CI)	OS HR (95% CI)	Effect
Gadgeel SM et al., 2020 (KEYNOTE-189) [53]	36 ICI/18 chemo arm	Stage IV; pembro + chemo vs. chemo	Not reported	Reported (co-mut subgroup)	Lower in mSTK11	mSTK11: 0.81 (0.44–1.47); wt: 0.38 (0.27–0.52)	mSTK11: 0.75 (0.37–1.50); wt: 0.59 (0.41–0.85)	Mixed
Cho BC et al., 2021 (KEYNOTE-042) [52]	16 ICI/17 chemo arm	Stage IV; pembro vs. chemo	Not reported	Reported	Similar	mSTK11: 0.75 (0.36–1.57)	mSTK11: 0.37 (0.16–0.86)	No effect
Papillon-Cavanagh S et al., 2020 [64]	574	Stage IV; real-world 1L ICI	Not reported	Reported; not predictive	Lower (mSTK11: NR)	mSTK11 vs. wt: 1.05 (0.76–1.44)	mSTK11 vs. wt: 1.13 (0.76–1.67)	No effect
Skoulidis F et al., 2018 (CheckMate-057/real-world) [8]	174 KRAS-mut	Stage IV; ICI (KRAS-mut subgroup)	KRAS-mutant (all)	Not stratified	mSTK11/KRAS: 7.4% vs. KRAS-only: 28.6%	mSTK11 vs. wt: 1.87 (1.32–2.66)	Shorter (*p* = 0.0015)	Negative
West HJ et al., 2022 (IMpower150) [55]	113 KRAS-mut with STK11/KEAP1	Stage IV; ABCP vs. ACP vs. BCP	KRAS-mutant (stratified)	*STK11*+/*KEAP1* co-mut.	NR	ABCP vs. ACP: HR 0.49 (0.28–0.84)	ABCP vs. ACP: HR 0.60 (0.34–1.03)	Mixed
Ricciuti et al., 2022 [7]	260 KRAS-mut	Stage IV; ICI (KRAS-mut)	KRAS-mutant (all)	*STK11*+*KEAP1* co-mut subgroup	mSTK11: 11.6% vs. wt: 32.4%	mSTK11 vs. wt: 2.04 (1.66–2.51)	mSTK11 vs. wt: 2.09 (1.68–2.61)	Negative
Skoulidis F et al., 2024 [48]	439 KRAS-mut	Stage IV; ICI + chemo (KRAS-mut)	KRAS-mutant (all)	*STK11*+*KEAP1* co-mut subgroup	NR	mSTK11 vs. wt: 1.60 (1.24–2.07)	mSTK11 vs. wt: 1.55 (1.18–2.05)	Negative
Sun L et al., 2024 [54]	2593	Stage IV; ICI ± chemo	KRAS-mutant (stratified)	Not reported	NR	NR	mKRAS/mSTK11 vs. wt/wt: HR 2.37 (1.34–2.75)	Negative

Abbreviations: NSCLC = Non-small cell lung cancer; ICI = Immune Checkpoint Inhibitor; PFS = Progression-Free Survival; OS = Overall Survival; HR = Hazard Ratio; CI = Confidence Interval; NR = Not reported; ABCP: Atezolizumab + Bevacizumab + Carboplatin + Paclitaxel; ACP: Atezolizumab + Carboplatin + Paclitaxel. Color Legend: 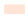 Significant negative effect of STK11 mutation on ICI outcomes; 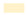 Mixed results or significant only in a specific subgroup/arm; 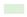 No significant negative effect of STK11 mutation detected.

**Table 3:** Emerging therapeutic strategies in STK11/KEAP1 mutant NSCLC.

Mechanism Category	Pathway	Primary Target	Drug	Trial Phase	Treatment Backbone	NCT number	Status
Immune resistance reversal	AXL pathway inhibition	AXL	bemcentinib	Phase1b/2a	Pembrolizumab + pemetrexed + carboplatin	NCT05469178	Terminated (lack of efficacy)
Metabolic–immune reprogramming	Polyamine synthesis inhibition	ODC1	DFMO	Phase 1/2	Pembrolizumab	NCT06219174	Suspended (drugs unavailable)
Innate immune modulation	Complement pathway	CFH	GT103	Phase 2	Pembrolizumab	NCT07017829	Recruiting
Epigenetic priming	CoREST inhibition	CoREST/HDAC	TNG260	Phase 1/2	Pembrolizumab	NCT05887492	Recruiting
Immune vulnerability	CD38 targeting	CD38	daratumumab	Phase 2	Monotherapy	NCT05807048	Recruiting
Metabolic vulnerability	Glutamine synthesis inhibition	Glutaminase inhibitor	telagelenstat	Phase 1	Monotherapy	NCT03872427	Active, not recruiting
Metabolic vulnerability	Glutamine synthesis inhibition	Glutaminase inhibitor	telagelenstat	Phase 2	Pembrolizumab + chemotherapy	NCT04265534	Terminated (lack of efficacy)
Metabolic vulnerability	mTOR suppression	mTROC 1/2 inhibitor	onatasertib	Phase 1	Monotherapy	NCT04518137	Terminated
Metabolic vulnerability	NFE2L2 pathway	NFE2L2/*KEAP1*/CUL3	MGY-825	Phase 1	Monotherapy	NCT05275868	Terminated
Metabolic vulnerability	*KEAP1* pathway	*KEAP1* activator	VVD-130037	Phase 1/2	Monotherapy or combination with chemotherapy or immunotherapy	NCT05954312	Recruiting

Abbreviations: NSCLC, Non-small cell lung cancer; STK11, serine/threonine kinase 11; *KEAP1*, Kelch-like ECH-associated protein 1; ODC1, ornithine decarboxylase 1; CFH, complement factor H; HDAC, histone deacetylase; CoREST, corepressor for element-1-silencing transcription factor; AXL, AXL receptor tyrosine kinase; mTOR, mechanistic target of rapamycin; NFE2L2, nuclear factor erythroid 2–related factor 2; CUL3, cullin 3.

## Data Availability

No new data were created or analyzed in this study. Data sharing is not applicable to this article.

## Abbreviations

The following abbreviations are used in this manuscript:

ABCP	Atezolizumab + Bevacizumab + Carboplatin + Paclitaxel
ACP	Atezolizumab + Carboplatin + Paclitaxel
AKT	Protein Kinase B
ALK	Anaplastic Lymphoma Kinase
AMPK	AMP-Activated Protein Kinase
ATR	Ataxia Telangiectasia and Rad3-Related Protein
AXL	AXL Receptor Tyrosine Kinase
BRAF	v-Raf Murine Sarcoma Viral Oncogene Homolog B
BCP	Bevacizumab + Carboplatin + Paclitaxel
CD38	Cluster of Differentiation 38
CDKN2A	Cyclin-Dependent Kinase Inhibitor 2A
CFH	Complement Factor H
CI	Confidence Interval
CR	Complete Response
CRT	Chemoradiotherapy
CTLA-4	Cytotoxic T-Lymphocyte–Associated Protein 4
DDR	DNA Damage Response
DFS	Disease-Free Survival
DNMT1	DNA Methyltransferase 1
DFMO	Difluoromethylornithine
EGFR	Epidermal Growth Factor Receptor
EMT	Epithelial–Mesenchymal Transition
EZH2	Enhancer of Zeste Homolog 2
FAK	Focal Adhesion Kinase
FDA	Food and Drug Administration
HIF1A	Hypoxia-Inducible Factor 1-Alpha
HR	Hazard Ratio
ICI	Immune Checkpoint Inhibitor
IHC	Immunohistochemistry
IL-1α/IL-33	Interleukin-1α/Interleukin-33
KEAP1	Kelch-Like ECH-Associated Protein 1
KRAS	Kirsten Rat Sarcoma Viral Oncogene Homolog
LKB1	Liver Kinase B1 (gene product of STK11)
LOH	Loss of Heterozygosity
LUAD	Lung Adenocarcinoma
MET	Mesenchymal–Epithelial Transition Factor
MO25	Mouse Protein 25
mRNA	Messenger Ribonucleic Acid
mTOR	Mechanistic Target of Rapamycin
mTORC1/2	mTOR Complex 1/Complex 2
NCCN	National Comprehensive Cancer Network
NFE2L2/NRF2	Nuclear Factor Erythroid-2 Related Factor 2
NGS	Next-Generation Sequencing
NSCLC	Non-Small Cell Lung Cancer
ODC1	Ornithine Decarboxylase 1
ORR	Objective Response Rate
OS	Overall Survival
P53/TP53	Tumor Protein 53
PD	Progressive Disease
PD-1	Programmed Cell Death Protein-1
PD-L1	Programmed Death-Ligand 1
PFS	Progression-Free Survival
PI3K	Phosphoinositide 3-Kinase
PR	Partial Response
PROTAC	Proteolysis-Targeting Chimera
PTEN	Phosphatase and Tensin Homolog
RET	Rearranged During Transfection
ROS1	ROS Proto-Oncogene 1
SAMe	S-Adenosyl-Methionine
SD	Stable Disease
SMARCA4	SWI/SNF-Related Matrix-Associated Actin-Dependent Regulator Subfamily A Member 4
STING	Stimulator of Interferon Genes
STK11	Serine/Threonine Kinase 11
STRAD	STE20-Related Adaptor Protein
TCR	T-Cell Receptor
TGF-β	Transforming Growth Factor Beta
TH1	T-Helper Cell Type 1
TILs	Tumor-Infiltrating Lymphocytes
TMB	Tumor Mutational Burden
TME	Tumor Microenvironment
VEGF	Vascular Endothelial Growth Factor
